# Neurobiological insights into perinatal depression: a multi-dimensional integration of brain structure, function, and metabolism

**DOI:** 10.3389/fpsyt.2026.1802534

**Published:** 2026-04-01

**Authors:** Yue Sun, Lanqing Liu, Chaozhi Bu, Minhui Jiang, Xiaomin Zheng

**Affiliations:** 1 Wuxi Maternity and Child Health Care Hospital, Affiliated Women’s Hospital of Jiangnan University, Wuxi, China; 2 Department of Acupuncture and Massage, The First People’s Hospital of Xuzhou, Xuzhou, China

**Keywords:** amygdala, brain metabolism, default mode network, hippocampus, perinatal depression, prefrontal cortex

## Abstract

**Objective:**

Perinatal depression (PND) represents a significant challenge to maternal and infant health. In recent years, the rapid advancement of neuroimaging techniques has offered critical insights for elucidating the neurobiological mechanisms underlying PND. This review aims to systematically synthesize the research progress on structural, functional, and metabolic abnormalities in specific brain regions of patients with perinatal depression, as well as the underlying pathological mechanisms, thereby providing a theoretical basis for early screening of PND.

**Methods:**

A systematic search was conducted in the PubMed and Web of Science databases for studies published up to January 2026, including those that compared characteristics between patients with perinatal depression and healthy controls.

**Results:**

Perinatal depression is a disorder with a robust neurobiological foundation, characterized by structural and functional alterations, as well as metabolic imbalances in key brain regions. These are manifested as reduced activity in regulatory centers and hyperactivity in emotional processing areas. Such abnormalities are the result of a complex interplay among genetic, neuroendocrine, immunological, and environmental factors.

**Conclusion:**

PND involves complex dysfunction within emotional and cognitive networks. These findings enhance the understanding of its neural basis and may inform future diagnostic and therapeutic strategies.

## 1 Introduction

The perinatal period represents a complex and highly vulnerable transition to motherhood. Perinatal depression (PND) is defined as a minor or major depressive episode occurring during pregnancy or within the first 12 months postpartum, encompassing both antenatal depression (AND) and postpartum depression (PPD) (1). The global prevalence of PND stands at 11.9%, with rates in low- and middle-income countries (13.1%) widely acknowledged to be higher than those in high-income countries (11.4%) (2). As one of the most common complications affecting women during the perinatal period, it has emerged as a significant public health burden worldwide in recent years. PND severely compromises the health of both mothers and their offspring. Studies have found that the suicide rate within one year postpartum among mothers with postpartum psychiatric disorders is significantly higher than that among healthy mothers (3), establishing PND as a major risk factor for maternal suicide. Furthermore, affected pregnancies are often accompanied by obstetric complications such as gestational hypertension, placental abruption, and postpartum hemorrhage (4), which increase the risk of adverse birth outcomes in offspring. Compared to children of mothers without perinatal depression, those exposed to maternal depression face significantly elevated risks of low birth weight, cesarean section (OR = 1.154), and preterm birth (OR = 1.404) (5, 6). Of greater concern, the impact of perinatal depression on offspring extends into childhood and adolescence, children exposed to maternal perinatal depression and anxiety exhibit a significantly higher risk of impairments in cognitive, emotional, and behavioral development (7, 8). This underscores the critical importance of screening and intervention during the unique time window of the perinatal period. In light of this, PND has evolved beyond the scope of a mere psychiatric disorder into an interdisciplinary issue integrating maternal-fetal medicine, pediatrics, mental health, and public health.

Current investigations into the pathological mechanisms of PND have advanced from classical neuroendocrine and neurotransmitter hypotheses—such as hypothalamic-pituitary-adrenal axis dysfunction and monoamine imbalances—to a more sophisticated focus on brain structures and neural circuits (9). This paradigm shift highlights a distinct neurobiological foundation for the disorder, characterized by glutamatergic pathway dysregulation, neural damage, and altered connectivity (10). As a direct proxy for neuronal energy expenditure and functional activity, brain metabolism has emerged as a critical window into the underlying pathophysiology. Advancements in modern neuroimaging, including functional magnetic resonance imaging (fMRI), magnetic resonance spectroscopy (MRS), and positron emission tomography (PET), have provided essential tools for interrogating brain function and metabolic status. However, existing research is often constrained by small sample sizes and high heterogeneity. Due to technical and ethical limitations, clinical biomarkers with diagnostic specificity remain elusive. Furthermore, current evidence remains fragmented, lacking a unified framework that integrates the “hormone-immune-gut-brain” axis with region-specific brain metabolism. This review systematically examined studies on the structural, functional, and metabolic alterations in brain regions associated with PND and summarized the underlying neuropathophysiological mechanisms. The findings aim to deepen the understanding of the neurobiological underpinnings of PND and provide a theoretical foundation for advancing its objective diagnosis and targeted therapy.

## 2 Methods

### 2.1 Search strategy

We systematically searched the PubMed and Web of Science databases for articles published in English up to January 2026, without any date restriction. In all databases, the search strategy combined MeSH terms and free-text words. The search terms included: (“Perinatal Depression” or “Antenatal Depression” or “Postpartum Depression” or “Pregnancy” or “Mothers” or “Depressive Disorder”), (“Nervous System” or “Neurobiology”), (“Brain Morphology” or “Hippocampus” or “Prefrontal Cortex” or “Amygdala”), (“Brain Metabolism” or “Brain Function”), and (“Functional Magnetic Resonance Imaging” or “fMRI” or “Positron-Emission Tomography” or “PET” or “Magnetic Resonance Spectroscopy” or “MRS”).

### 2.2 Inclusion and exclusion criteria

Inclusion criteria were as follows: The study population included women diagnosed with perinatal depression during pregnancy or within the first 12 months postpartum, with a control group consisting of women without perinatal depression during the same period; Diagnosis of perinatal depression was confirmed using structured clinical interviews or other standardized diagnostic instruments. Exclusion criteria were as follows: Meta-analyses, systematic reviews, or non-systematic reviews; Studies using non-validated methods to diagnose perinatal depression; Studies with assessment time points beyond 12 months postpartum; Absence of a control group.

The following were considered as potential confounding factors but were not reasons for exclusion: medication use; comorbid anxiety disorders; first episode or previous history of depression; socioeconomic status; educational level; and number of prior pregnancies.

### 2.3 Study selection and data extraction

**Figure 1:**
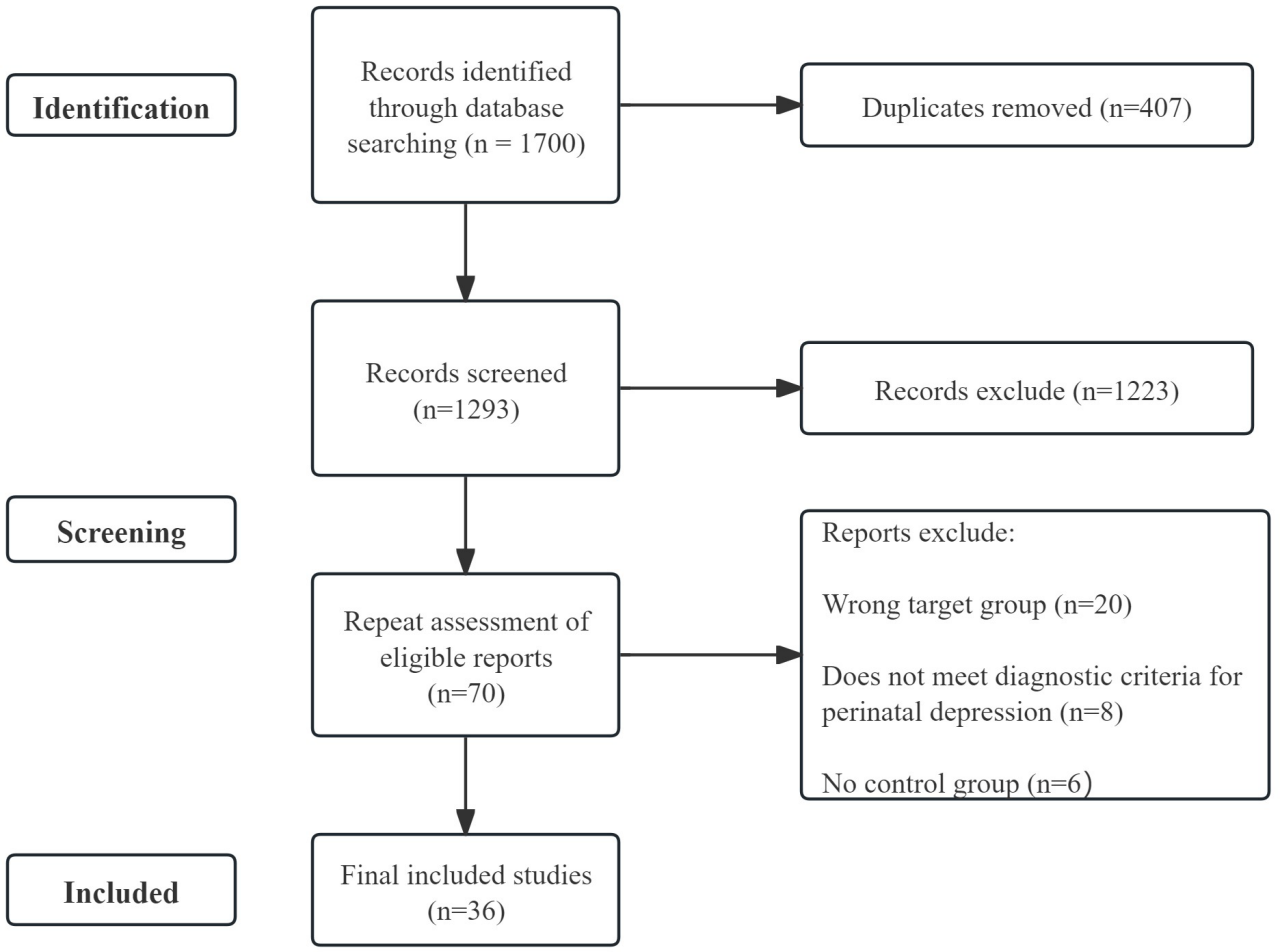
Flowchart of the study selection process. A total of 1, 700 records were initially identified from PubMed and Web of Science. After the removal of 407 duplicate records and the exclusion of 1, 223 articles during title and abstract screening, 70 reports were assessed for eligibility. Following a full-text review, 34 reports were excluded for specific reasons (wrong target group, n = 20; non-standard diagnostic criteria, n = 8; and absence of a control group, n = 6). Finally, 36 studies were included in this systematic review.

Two authors independently screened the literature, extracted data, and cross-checked the results. Any disagreements were resolved through discussion or by consultation with a third reviewer. During the literature screening process, duplicate records were first removed, followed by an initial screening based on titles and abstracts. Subsequently, full-text reviews were conducted to determine the final inclusion of studies. The detailed literature screening and selection process, including reasons for exclusion, is illustrated in the flowchart (**Figure 1**). Data extraction was subsequently performed, encompassing the following items: first author and year of publication; study design; sample size; assessment of depressive symptoms; and key findings related to neural features. Quality assessment of each included study was independently conducted by two authors. Consensus between the two authors was required for final inclusion of studies in the systematic review.

### 2.4 Analysis and synthesis of the results

Due to the substantial heterogeneity across the included studies, a narrative synthesis approach was employed when systematically comparing the structural, functional, and metabolic brain alterations between women with and without PND.

## 3 Structural, functional and metabolic abnormalities in brain regions

Previous studies have revealed that pregnant women exhibit an overall reduction in brain volume (11), characterized specifically by decreased gray matter volume (GMV) in the anterior and posterior midline cortical regions, as well as the bilateral lateral prefrontal and temporal cortices (12). The development of PND is intricately linked to key brain regions involved in emotion regulation and self-referential processing, including the prefrontal cortex, amygdala, hippocampus, anterior cingulate cortex, and the default mode network. In this section, we systematically delineate the structural alterations, functional disruptions, and metabolic abnormalities observed within these regions.

### 3.1 Prefrontal cortex

The prefrontal cortex (PFC) serves as a cornerstone for human cognitive functions, emotional regulation, social capabilities, and motivational drive (13). A wealth of evidence indicates that PFC dysfunction is a common denominator in various psychiatric conditions, including depression and schizophrenia. Within this architecture, the dorsolateral prefrontal cortex (DLPFC) and ventromedial prefrontal cortex (VMPFC) are pivotal as hubs for higher-order cognition and affective control (14). Furthermore, the medial prefrontal cortex (MPFC) plays a vital role in the neural circuitry modulating the hypothalamic pituitary adrenal (HPA) axis and emotional responses to stress.

At the macroscopic structural level, reduced volumes in the hippocampus and the medial prefrontal cortex are consistently identified in MDD (15). However, structural alterations in PND appear to be more complex. One MRI study reported significantly increased regional GMV in the DLPFC and right anterior insula in patients with PPD (16). Another similar investigation observed comparable changes, suggesting that increased GMV in the DLPFC may represent a distinct pathological structural adaptation in response to stress in PPD patients (17). Conversely, another study by Schnakenberg et al. found no significant structural or functional brain differences between PPD patients and healthy controls (18). This inconsistency may be attributed to methodological heterogeneity, such as variations in sample size and differences in neuroimaging and analytical techniques. Notably, the pattern of GMV increases observed in PPD stands in stark contrast to the GMV reductions commonly found in the medial, ventral, and dorsal prefrontal systems (including the anterior cingulate cortex) in patients with major depressive disorder (19, 20) GMV reduction is commonly observed in multiple psychiatric disorders (e.g., Alzheimer’s disease, major depressive disorder), reflecting neuronal loss and functional abnormalities. Conversely, the increased GMV observed in PPD may suggest an association with neuroinflammatory processes in the central nervous system. This distinct neurobiological alteration warrants further investigation as a potential biomarker for PPD.

In animal models, the PFC also demonstrates stress-induced microstructural changes; for instance, chronic mild stress during the postpartum period induces dendritic spine loss in the MPFC of rats (21), while prenatal stress exposure reduces spine density and alters the morphology of pyramidal neurons in the same region (22). These findings suggest that chronic stress impairs neural connectivity and plasticity within the PFC, driving the progression of PND.

Regarding functional alterations, PPD patients exhibit significant abnormalities in spontaneous neural activity. Elevated activity in the DLPFC has been linked to impaired emotional regulation, while significantly lower regional homogeneity (ReHo) between the DLPFC and the insula indicates reduced local neural synchronization. Additionally, the fractional amplitude of low-frequency fluctuations (fALFF) in the DLPFC is markedly elevated (23, 24), a phenomenon also observed in the left MPFC of untreated mothers with AND (25). Further research reveals reduced voxel-mirrored homotopic connectivity (VMHC) in the bilateral dorsomedial prefrontal cortex, dorsal anterior cingulate cortex, and orbitofrontal cortex in PPD patients. Specifically, VMHC values in the dorsomedial prefrontal cortex (DMPFC) correlate negatively with Edinburgh postnatal depression scale (EPDS) scores (24). Moreover, weakened functional connectivity between the anterior cingulate cortex (ACC), amygdala, and DLPFC further compromises cognitive emotional regulation in PPD (26). The aforementioned functional connectivity abnormalities in regions such as the prefrontal cortex may impair cognitive and emotional regulation, triggering negative emotions and interoceptive discomfort, thereby contributing to the pathological progression of perinatal depression.

Neurotransmitters are core chemical messengers that regulate neural function, and their metabolic imbalance represents a central manifestation of regional cerebral metabolic abnormalities. Although the serotonin (5-HT) hypothesis remains a subject of ongoing debate, 5-HT modulators continue to be a clinical mainstay (27). In PPD patients, studies have identified a 34% increase in monoamine oxidase A (MAO-A) density within the ACC and PFC (28), with more extensive distribution observed in patients prone to excessive crying (29), potentially leading to depleted local 5-HT levels.

Concerning amino acid neurotransmitters, the equilibrium between excitatory glutamate (Glu) and inhibitory γ-aminobutyric acid (GABA) is critical. In patients with MDD, brain regions exhibit decreased glutamate levels and a 67% increase in metabotropic glutamate receptor 2/3 expression in the PFC (30, 31). Hu et al. reported similar findings in patients with PPD (32). In animal models, it was also observed that gestational stress-induced rats exhibited impaired reactivity of the glutamatergic system in the prefrontal cortex (33). Additionally, in a learned helplessness (LH) rat model, amino acid metabolic imbalances—including disruptions in glutamate and arginine metabolism—were identified in the same brain region (34). Interestingly, some PPD studies have noted elevated glucose and glutamate levels in the MPFC (35). These divergent results may reflect the sensitivity of the glutamatergic system to the dramatic ovarian hormone fluctuations characteristic of the postpartum period. Furthermore, significant reductions in Glu + glutamine (Glx) and N-acetylaspartic acid (NAA) + N-acetylglutamine (NA) have been recorded in the DLPFC of PPD groups (36). The decline in NAA, similar to findings in chronic MDD (37), is echoed in chronic social defeat stress (CSDS) models (38). Conversely, MDD is associated with increased GABAergic signaling in the lateral prefrontal cortex (39). Collectively, these findings highlight a complex and heterogeneous dysregulation across multiple neurotransmitter systems in PND.

### 3.2 Anterior cingulate cortex

The anterior cingulate cortex (ACC), a pivotal component of the limbic system, is functionally bifurcated into the ventral ACC and dorsal ACC. This region governs emotion regulation, cognitive control, pain processing, and the modulation of empathetic behaviors (40). Consequently, its dysfunction is inextricably linked to the cognitive and affective deficits observed in depression (41).

At the structural level, studies on structural and functional changes in the ACC among individuals with PND remain relatively limited. In contrast, research on MDD has consistently revealed reductions in GMV and white matter microstructural abnormalities (e.g., reduced fractional anisotropy) in the ACC among both elderly and adolescent patients (42, 43). Furthermore, depressed patients with suicidal tendencies exhibit significantly fewer dendritic branches in pyramidal neurons of the anterior cingulate cortex (44). However, whole-brain MRI studies focusing on PPD have not observed significant gray or white matter volume changes in the ACC (15), which may suggest that the macrostructural and microstructural alterations in PND are more subtle compared to those in typical MDD.

Functional neuroimaging studies have further revealed abnormal activity patterns in the ACC among individuals with PND. One study reported reduced activity in the left ACC in PPD patients (17, 45), and this decreased activity was accompanied by altered connectivity with other brain regions (e.g., the amygdala), which may mediate emotional disturbances and somatic symptoms (46). On the other hand, PPD patients with comorbid anxiety exhibited significantly increased amplitude of fALFF in the subgenual anterior cingulate cortex during the resting state, compared to those with PPD alone and healthy controls (47). Animal experiments have further confirmed impaired resting-state functional connectivity in the ACC in a postpartum rat model of chronic social stress (48). Additionally, disrupted functional connectivity between the posterior cingulate and the amygdala has been observed in women with PPD (49), along with alterations in gray matter structural covariance networks. The ACC, as a key brain region involved in emotional regulation, may also exhibit changes in its structural covariance networks, which may indirectly affect its connections with other brain regions and impair an individual’s ability to regulate cognition and emotions (50).

At the metabolic level, in addition to the significantly increased MAO-A density in the PFC and ACC mentioned earlier, PPD patients specifically exhibit significantly lower postsynaptic 5-HT1A receptor binding in the ACC and adjacent temporal cortex (51), this region’s 5-HT1A receptors regulate neural circuits involved in emotion regulation, suggesting that decreased 5-HT1A receptor binding may mediate the development and progression of anxiety and depressive symptoms in postpartum women. Additionally, increases in GABA- and glutamate-related genes in the ACC may be associated with suicidal behavior (52). In adolescent patients with MDD, particularly those with comorbid anhedonia, significantly reduced GABA levels have also been observed in the ACC. Interestingly, unlike the glutamatergic dysfunction in MDD, the glutamatergic system in the ACC of patients with PPD may be susceptible to hormonal fluctuations. In the context of a sharp decline in estrogen and progesterone levels during the postpartum period, no significant glutamatergic dysfunction was detected in the anterior cingulate cortex of PPD patients using 1H-MRS. However, increased levels of glutamate and the sum of glutamate and glutamine were observed in individuals using progestin-based contraceptives (36). This finding may suggest that dramatic perinatal hormonal fluctuations (particularly progesterone) could increase maternal susceptibility to mood disorders by affecting glutamatergic neurotransmission.

### 3.3 Amygdala

The amygdala, situated within the medial temporal lobe, is a compact subcortical structure primarily subdivided into the basolateral amygdala and the central medial amygdala. It functions as a pivotal hub for socioemotional and cognitive integration and constitutes a core node within emotion-related neural circuitry (53).

Investigations into structural alterations of the amygdala and its subnuclei in MDD have yielded inconsistent findings. For instance, Kim et al. reported an enlargement of the amygdala and its subregions in MDD patients, whereas Okamoto et al. failed to observe such changes (54, 55). Other evidence suggests that stress levels correlate positively with the volume of the right amygdala and anterior hippocampus, with depression exhibiting a particularly robust association with right amygdala volume (56). Regarding PPD, studies have indicated potential structural impairments; specifically, resting-state functional MRI scans revealed significantly reduced GMV in the bilateral lateral amygdala, though this finding may be influenced by limited sample sizes. Notably, while some data show no significant differences in the resting-state functional connectivity (RSFC) of amygdala subregions between PPD patients and healthy females, Tang et al. observed no discernible differences in amygdala GMV between patients with general depression and healthy controls (57). In preclinical models, gestational stress in rats has been shown to increase dendritic spine density in the basolateral amygdala postpartum—a structural shift that appears resistant to selective serotonin reuptake inhibitors (58).

In cases of severe PPD, diminished coupling between the posterior cingulate cortex and the right amygdala has been documented, suggesting that the pathophysiology of PPD may involve disruptions in self-referential processing and empathy (49). While Habas et al. proposed that the cerebello-amygdala network is essential for sensorimotor, emotional, and motivational integration (59), rs-fMRI data from depressed patients have revealed attenuated connectivity within this circuit, potentially reflecting a broader imbalance in network homeostasis (60). Furthermore, functional neuroimaging in postpartum women has demonstrated that those with PPD exhibit blunted amygdala reactivity to external stimuli (61). This is corroborated by Laurent et al., who identified impaired amygdala activation in mothers with PPD—a deficit potentially linked to compromised mother-infant bonding and maladaptive caregiving, such as delayed responsiveness to infant distress (62). Interestingly, Dudin et al. found that mothers with PPD exhibit a more pronounced amygdala response to images of infant smiles compared to women with general depression, yet remain unresponsive to emotionally positive but neutral stimuli (63).

Metabolic dysregulation within the amygdala is a hallmark of various psychiatric disorders, yet research specifically addressing PND remains sparse. In general depression, altered amygdala glycolysis has been reported, and transcriptomic analysis by Zhang et al. revealed an upregulation of bioenergetic pathways in MDD patients, which may correlate with anxiety symptoms (64). Similar metabolic shifts are observed in both CSDS and CUMS rat models, characterized by significant perturbations in amino acid and lipid metabolism, a marked reduction in glutamate levels, and substantial changes in synapse-associated proteins. Collectively, these findings underscore the role of aberrant energy metabolism within the amygdala in the pathogenesis of depression (65).

### 3.4 Hippocampus

The hippocampus is essential for learning, memory, and spatial navigation, serving as a cornerstone of the limbic system. Substantial evidence links hippocampal dysfunction to various psychiatric conditions, including depression, schizophrenia, and anxiety disorders (66).

Notably, hippocampal atrophy remains the most consistently replicated structural finding in depression research. Detailed investigations into hippocampal subfields suggest their differential involvement in the progression of MDD, with the left CA1 subregion identified as a potential biomarker for the disorder (67). This is supported by findings of significant volume reduction in the left CA1 among MDD patients exhibiting anhedonia compared to healthy cohorts (68). In the context of PND, pregnant women with AND exhibit decreased fALFF in the parahippocampal gyrus, where fALFF values correlate negatively with EPDS scores (69). Complementary neuroimaging data from women with AND also reveal reduced ReHo within both the amygdala and hippocampus, which similarly shows an inverse correlation with EPDS scores (25). Furthermore, research has identified weakened functional connectivity between the right hippocampus and the right middle frontal gyrus in PPD patients. These results suggest that the right hippocampus plays a more prominent role in the neural network architecture of PPD, characterized by significantly reduced coupling with the DLPFC (70, 71), given the role of the hippocampus in stress regulation, functional connectivity disruptions in these brain regions may adversely affect maternal mental health outcomes. Koeppel et al. reported an intriguing finding: unlike in MDD, right hippocampal volume in patients with PPD was inversely associated with treatment outcome (72). This inverse association may provide supporting evidence for distinguishing PPD from MDD. As previously discussed, PPD likely stems from maternal hypersensitivity to hormonal fluctuations, along with distinct neuropathological alterations. In this context, reduced hippocampal volume may reflect a physiological adaptation that facilitates the transition to motherhood. However, given the relatively small sample size of this study, larger-scale longitudinal studies are warranted to validate this finding and explore the underlying pathophysiological mechanisms.

In contrast to the reduced dopamine levels typically associated with MDD, a mouse model of postpartum depression demonstrated low 5-HT and NE but high dopamine levels in the hippocampus, a profile that probably indicates reward system dysfunction (73). Additionally, chronic mild stress rat model exhibited neurotransmitter imbalances in the hippocampus, characterized by elevated levels of N-acetylaspartate and glutamate, alongside reduced levels of aspartate and GABA (74). The CUMS model also revealed significantly elevated glutamate levels in the hippocampus, a dysregulation that may mediate astrocyte apoptosis. Furthermore, in animal models of prenatal stress, increased glycolytic metabolism (as evidenced by phosphofructokinase) and reduced Krebs cycle activity (indicated by decreased pyruvate dehydrogenase) have been observed (75). This metabolic pattern may increase the risk of lactate accumulation, further compromising hippocampal neuronal integrity.

### 3.5 Default mode network

The default mode network (DMN) is an anatomically defined brain system characterized by heightened activity during resting states and is closely linked to self-referential thought, emotional rumination, and other internal psychological processes (76). Mirroring findings in MDD, enhanced functional activity within the DMN has also been observed in patients with PPD (10). Specifically, the DMPFC in PPD exhibits increased connectivity with other DMN components. However, Deligiannidis et al. identified reduced RSFC between the DMPFC and the precuneus, PCC, and supramarginal/angular gyrus regions in these patients. Such disrupted connectivity between the DMPFC and these posterior nodes may clinically manifest as deficits in self-evaluation and emotional perception (71). At the neurochemical level, GABAergic signaling is notably diminished within DMN regions (39, 77). Furthermore, PPD involves a broad network of regions responsible for integrating emotion and cognition. Significant alterations in information flow patterns have been documented across the amygdala, cingulate cortex, insula, hippocampus, and the frontal, parietal, and occipital lobes (78), suggesting that impaired inter-regional coordination across global brain networks is a fundamental component of the neural mechanisms underlying perinatal depression.

Overall, the neurobiological landscape of PND is characterized by a complex array of alterations spanning from macroscopic morphology and network connectivity to microscopic molecular metabolism. These multidimensional findings—encompassing structural atrophy, functional dysconnectivity, and neurochemical imbalances across key emotional and cognitive brain hubs—are integrated and summarized in **Tables 1**–**3**.

**Table 1:** Structural changes in brain regions.

Brain region	Types of depression	Macrostructural changes	Study	Types of depression	Microstructural changes	Study
Prefrontal Cortex	MDD	Hippocampus and MPFC volume decreases.	Belleaue et al., 2019 (15)	PPD	MPFC dendritic spines shed	Michelsen et al., 2007 (21)
	MDD	GMV decreases in medial, ventral and dorsal prefrontal systems	Pizzagalli et al., 2022 Wise et al., 2017 (19, 20)	AND	Dendritic spine density of pyramid neurons in MPFC	Woo et al., 2021 (22)
	PPD	GMV significantly increased in the left DLPFC and the right anterior insula.	Cheng et al., 2022. Chen et al., 2023 (17, 47)			
	PPD	No difference.	Schnakenberg et al., 2021 (18)			
Anterior Cingulate Cortex	MDD	The GMV in the ACC region decreases.	Harada et al., 2018 (42)	MDD	Pyramidal neurons in the anterior cingulate cortex exhibit a significant reduction in the number of dendritic branches.	Hercher et al., 2010 (44)
	MDD	ACC white matter decreased significantly.	Gabbay et al., 2012 (43)			
	PPD	ACC did not observe changes in gray matter and white matter.	Belleau et al., 2019 (15)			
Amygdala	MDD	Amygdala volume increases.	Kim et al., 2021 (54)	AND	Stress increases spine density in the BLA and this change cannot be reversed by SSRI	Haim et al., 2016 (58)
	PPD	GMV of left lateral amygdala and right lateral amygdala decreased significantly; RSFC in amygdala subregions of PPD patients was no different from healthy women.	Huang et al., 2023 (57)			
Hippocampus	PPD	The volume of right hippocampus was inversely related to the effect of depression treatment.	Koeppel et al., 2026 (72)			
Default Mode Network				PPD	DMN functional activity is enhanced.	

**Table 2:** Changes in brain functional connectivity.

Brain region	Types of depression	Participants (n = numbers)	Functional changes	Study
Prefrontal Cortex	PPD	PPD(n=16) HPW(n=16)	Spontaneous neural activity increases in the DLPFC region.	Che et al., 2020 (23)
	PPD	PPD(n=28) HPW(n=28)	The ReHo between the DLPFC and the insula is significantly decreased, while the fALFF in the DLPFC is significantly increased.	Li et al., 2022 (24)
	AND	AND(n=21) HPW(n=22)	ReHo decreases in the left dorsolateral prefrontal cortex, right insular lobe, and right ventral temporal cortex, amygdala, and hippocampus.	Cheng et al., 2021 (25)
	PPD	PPD(n=20) HPW(n=12)	Functional connectivity between ACC, amygdala and DLPFC regions is weakened.	Deligiannidis et al., 2013 (26)
Anterior Cingulate Cortex	PPD	PPD(n=288) HPW(n=279)	Activity in the left anterior cingulate gyrus decreases.	Qiu et al., 2025 (45)
	PPD	PPD(n=45) HPW(n=62)	Abnormal resting state functional activity in the SGACC.	Cheng et al., 2022 (47)
	PPD	PPD(n=14) HPW(n=23)	Functional connectivity between the posterior cingulate cortex and the amygdala shows abnormal disruption.	Chase et al., 2014 (49)
Amygdala	PPD	PPD(n=14) HPW(n=23)	Coupling between the posterior cingulate cortex and the right amygdala is reduced.	Chase et al., 2014 (49)
	MDD	MDD(n=70) HPW(n=70)	Connectivity between the amygdala and the cerebellum is decreased.	Tang et al., 2019 (60)
	PPD	PPD(n=28) HPW(n=17)	PPD patients exhibit a weakened response of the amygdala to stimuli.	Wonch et al., 2016 (61)
Hippocampus	AND	AND(n=20) HPW(n=22)	The fALFF in the parahippocampal gyrus is decreased, and the fALFF values are negatively correlated with the EPDS scores.	Cheng et al., 2020 (69)
	AND	AND(n=21) HPW(n=22)	The ReHo of the hippocampus is decreased, and the ReHo values are also negatively correlated with the EPDS scores.	Cheng et al., 2020 (69)
	PPD	PPD(n=29) HPW(n=30)	The connectivity between the right hippocampal region and the right middle frontal gyrus is decreased.	Zhang et al., 2022 (70)

**Table 3:** Changes in brain metabolism.

Brain region	Types of depression	Metabolic changes	Study
Prefrontal Cortex	MDD	67% increase in metabotropic glutamate receptor 2/3 levels in PFC	Feyissa et al., 2010 (31)
	MDD	Increased GABAergic signaling is observed in the LPFC region.	Moriguchi et al., 2019 (39)
	PPD	MAO-A is significantly increased in the PFC region.	Zhang et al., 2024 (28)
	PPD	Patients with PPD who are prone to crying exhibit a broader distribution of MAO-A in the PFC and ACC.	Sacher et al., 2015 (29)
	PPD	Blood glucose and glutamate levels are elevated in the MPF region.	McEwen et al., 2012 (35)
	PPD	The levels of Glu + Glx and NA + NAA are significantly reduced in the DLPFC.	Rosa et al., 2017 (36)
Prefrontal Cortex	PPD	The density of MAO-A in the ACC is significantly increased compared to the healthy control group (by an average of 34%).	Zhang et al., 2024 (28)
	PPD	The postsynaptic 5HT1A receptors in ACC and near temporal cortex were significantly lower than those in healthy group.	Moses-Kolko et al., 2008 (51)
	PPD	No glutamatergic dysfunction was detected.	Rosa et al., 2017 (36)
Amygdala		Alterations in amino acid and lipid metabolism were observed, accompanied by a significant decrease in glutamate levels, along with notable changes in synaptic-associated proteins.	Li et al., 2021 (65)
Hippocampus	PPD	Neurotransmitter alterations manifest as low levels of 5-HT and NE, along with elevated levels of dopamine.	Avraham et al., 2017 (73)
Default Mode Network	PPD	GABAergic signaling is reduced in the DMN regions.	Horáková et al., 2022 (10)

## 4 Potential mechanisms of brain region abnormalities

**Figure 2:**
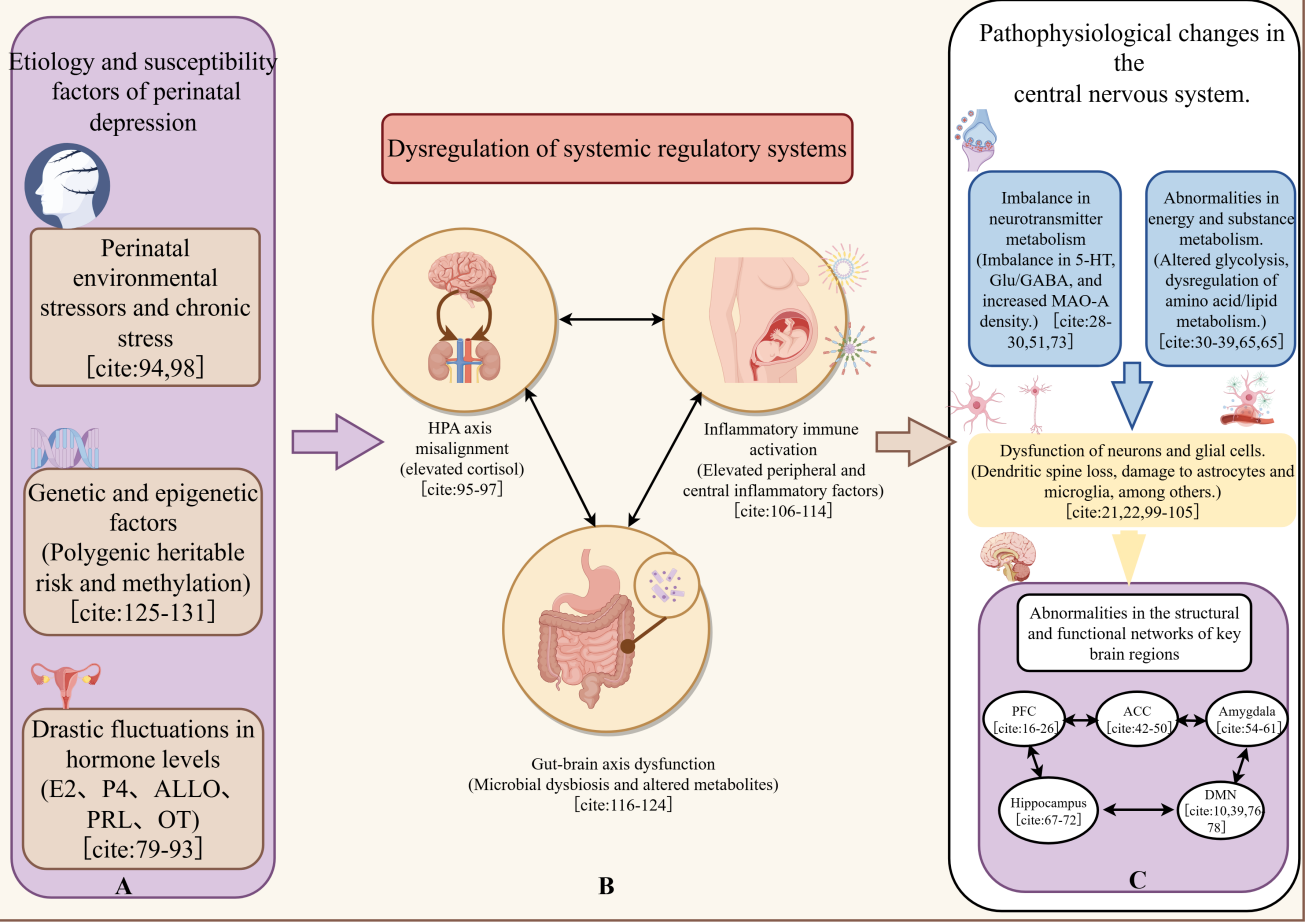
Schematic diagram of the multi-dimensional pathogenesis of perinatal depression (PND). The onset of PND is driven by the dynamic interaction between the “hormone-immune-gut-brain” axis and central neural activity. **(A)** Peripheral factors: Drastic fluctuations in estrogen, progesterone, and allopregnanolone trigger HPA axis hyperactivation and systemic inflammation. **(B)** Gut-brain axis: Microbiota dysbiosis influences brain function via neuroendocrine and metabolic pathways (e.g., tryptophan-serotonin metabolism). **(C)** Central alterations: These factors collectively lead to structural atrophy (e.g., hippocampal volume reduction), functional dysconnectivity (e.g., DMN hyperactivity), and metabolic dysregulation (e.g., glutamate/GABA imbalance) in emotion-regulation centers.

The neuropathophysiological framework of PND involves a complex interplay of multi-level factors, ranging from peripheral systemic shifts to central neurobiological alterations. As illustrated in **Figure 2**, dramatic hormonal fluctuations during the perinatal period serve as a primary trigger, which subsequently leads to HPA axis misalignment, inflammatory immune activation, and gut microbiota dysbiosis. These peripheral signals converge on key brain regions, such as the prefrontal cortex and limbic system, inducing structural remodeling, functional connectivity disruptions, and metabolic imbalances, which collectively contribute to the clinical manifestations of PND.

### 4.1 Hormonal fluctuations

The perinatal period represents the most dramatic phase of fluctuations in steroid and peptide hormones across a woman’s lifespan, and these fluctuations are closely associated with alterations in brain morphology, function, and neurotransmitter systems, particularly concerning the ovarian hormones estrogen (E2) and progesterone (P4). E2 and P4 receptors are widely distributed throughout the central nervous system, with brain regions integral to anxiety and depression—including the ACC, hippocampus, amygdala, and PFC—exhibiting heightened sensitivity to these fluctuations (79). Research has demonstrated that E2 exerts neurotrophic effects on the hippocampus, where synaptogenesis, GMV, and white matter integrity are positively correlated with E2 concentrations and negatively correlated with P4 levels (80); conversely, E2 concentrations show a significant negative correlation with GMV in the ACC and PFC (81). During pregnancy, estrogen and progesterone levels can reach up to 1, 000 times their normal values, followed by a sharp decline postpartum. This pattern suggests that the increased GMV in the PFC of PPD patients may be linked to the rapid withdrawal of E2 after childbirth, and that the direction of neuroplastic changes in PND differs from that in MDD, potentially due to direct effects of hormonal withdrawal (82). Furthermore, Mehta et al. identified differentially expressed transcripts between women with PPD and healthy controls, which were primarily enriched in estrogen signaling pathways (e.g., SP1 and TAF6) and predicted PPD with 88% accuracy (83). This suggests that genetic or epigenetic variations in the E2 signaling pathway may constitute a key determinant of individual susceptibility to PND, holding potential as biomarkers or therapeutic targets for PND. In animal studies, E2 has also been demonstrated to exert antidepressant and anxiolytic effects (84).

Furthermore, PND results from dysregulation of multiple hormonal systems, rather than being confined solely to ovarian hormones. The neuroactive steroid allopregnanolone (ALLO), a metabolite of P4, has emerged as a key player in the pathogenesis of mood disorders (85). The association between ALLO and affective symptoms fluctuates throughout the perinatal window; while no correlation is observed during late pregnancy, ALLO levels at six weeks postpartum are positively associated with both depression and anxiety (86). Longitudinal data from Björväng et al. further suggest that ALLO may exacerbate the duration of depressive symptoms (87). These effects likely stem from the dynamic reconfiguration of GABA_A_ receptors (GABA_A_-Rs) in response to neurosteroid shifts. Specifically, sustained elevations of neurosteroids during pregnancy trigger a down-regulation of the α5 and γ2 subunits of GABA_A_-Rs in the maternal cortex and hippocampus (88). Consequently, pathologically high ALLO levels may induce GABA_A_ receptor desensitization, thereby intensifying depressive or anxious phenotypes. Additionally, other peptide hormones contribute to this neurobiological framework: prolactin has been shown to safeguard hippocampal neurons and stimulate neurogenesis (89), yet its plasma levels are notably diminished in mothers with PPD (90). Similarly, multiple studies have established an inverse correlation between PND symptoms and oxytocin levels during late pregnancy or the postpartum period, with lower oxytocin levels linked to increased breastfeeding frustration (91). Indeed, high oxytocin concentrations may serve as a protective buffer against PND in high-risk populations (92). Finally, thyroid function also plays a role, as women with prenatal thyroxine levels below the physiological range exhibit increased vulnerability to postpartum depressive symptoms (93).

### 4.2 HPA axis misalignment

Dysregulation of the HPA axis remains one of the most consistent findings across diverse psychiatric research. During pregnancy, in addition to stress-induced HPA activation, the placenta—a unique gestational organ—synthesizes and secretes corticotropin-releasing hormone (CRH) into the maternal circulation. This precipitates significant elevations in maternal adrenocorticotropic hormone, cortisol, and placental corticotropin-releasing hormone (pCRH) levels (94). Evidence suggests that HPA axis dysfunction and the resulting hypercortisolemia are associated with diminished metabolite concentrations in the ACC (95). Clinical data further indicate that women exhibiting elevated pCRH levels during mid-gestation face a heightened risk of developing PPD at 2–3 months postpartum (96). Notably, while physiological levels of CRH can promote hippocampal synaptogenesis, pathological concentrations exert neurotoxic effects on hippocampal neurons. Furthermore, CRH crossing the placental barrier can adversely impact fetal neurodevelopment (97).

Glucocorticoid receptors (GRs) are extensively distributed within the hypothalamus, amygdala, hippocampus, MPFC, and ACC, where they mediate HPA axis feedback. Women with an EPDS score ≥10 at six weeks postpartum exhibit significantly higher cortisol levels than those with AND or healthy controls (98). Excessive cortisol exposure can cause structural damage to emotion-regulation hubs, particularly the hippocampus and PFC. In preclinical models, administration of high-dose corticosterone (CORT) to postpartum rats reduced dendritic complexity in the hippocampal CA3 region (99). In CORT-treated rats, neuronal atrophy and a marked reduction in spine density were observed in the nucleus accumbens, alongside similar spine density loss in pyramidal neurons of the hippocampal CA1 subregion (100). Additionally, CORT has been shown to induce oxidative stress in the murine hippocampus, activating the RIPK3 pathway, which impairs synaptic plasticity and triggers neuronal death (101). Beyond neuronal effects, chronic cortisol exposure can induce transcriptional reprogramming in astrocytes, disrupting their connectivity with neurons and further impairing synaptic plasticity. Given that astrocytes are vital for mood regulation and cerebral homeostasis, their functional impairment is considered a significant driver of depressive onset (102), with cortisol-induced astrocytic changes further exacerbating the severity of the disorder (103). Moreover, neuroinflammation and microglial hyperactivation play pivotal roles in the pathogenesis of depression; studies have confirmed that CORT modulates the activity of BV2 microglial cells (104). *In vitro* experiments using human hippocampal progenitor cells further demonstrate that dexamethasone treatment enhances neuroinflammatory responses, suggesting that cortisol may facilitate the progression of PND by intensifying inflammation within targeted brain regions (105).

### 4.3 Inflammatory immune activation

Accumulating evidence has identified elevated peripheral inflammatory markers in patients with PND. A large-scale Swedish prospective cohort analysis revealed that lower pre-pregnancy lymphocyte levels and a higher platelet-to-lymphocyte ratio are significant predictors of increased PND risk (106). Pro-inflammatory markers, including CRP, interleukin-1β (IL-1β), IL-6, and tumor necrosis factor-α (TNFα), have been extensively investigated and correlate with depressive symptom severity (107). Recent findings further indicate that levels of IL-6, IL-10, vascular endothelial growth factor, and TNFα during the third trimester are significantly elevated in women with severe prenatal depression compared to healthy cohorts (108), a result corroborated by Osborne et al. (109). Beyond peripheral shifts, patients with AND exhibit markedly higher concentrations of IL-1β, IL-23, and IL-33 in the cerebrospinal fluid, directly implicating central nervous system inflammation in the pathogenesis of the disorder (110). Furthermore, upregulated TNF expression during pregnancy is closely linked to the subsequent development of PPD (111). Studies focusing on the postpartum period also report elevated plasma IL-6 and IL-8 levels. Mechanistically, peripheral inflammation shunts tryptophan metabolism toward the kynurenine pathway, thereby depleting serotonin production in women with PPD (112). Since serotonin is essential for enhancing neuronal excitability and regulating calcium homeostasis in astrocytes, such inflammatory shifts may compromise the structural and functional integrity of targeted brain regions, exacerbating localized metabolic abnormalities (113). Additionally, the peripheral inflammatory response interacts synergistically with the HPA axis; in women with a family history of depression, each unit increase in the plasma IL-8/IL-10 ratio and salivary cortisol area under the curve was associated with a 1.50-fold and 2.16-fold increase in PPD incidence, respectively (114).

### 4.4 Gut-brain axis

A substantial body of research indicates a robust association between PND and alterations in the composition and function of the gut microbiota. The “gut-brain axis” concept, as postulated by Cryan et al., suggests that the gut microbiota interacts with the central nervous system through neuroendocrine, immune, and metabolic pathways, thereby modulating brain function and behavioral expression (115). Studies have demonstrated that diminished gut microbial diversity is linked to both maternal and infant anxiety and depressive symptoms (116). Specifically, prenatal EPDS scores correlate negatively with microbial population abundance (117), and significant shifts in fecal microbial composition are observed in women with AND, marked particularly by a relative depletion of beneficial genera such as Lactobacillus (118). A meta-analysis further indicated that perinatal probiotic supplementation effectively reduces depression scores (119). Additionally, Actinobacteria and the genus Holdemanella are considered to exert protective effects against PPD (120).

The “brain-gut axis” functions as a complex bidirectional feedback system that influences brain regions involved in central metabolism and emotional regulation through diverse mechanisms. On one hand, gut microbiota can metabolize dietary components into serotonin precursors (such as tryptophan) or directly stimulate enterochromaffin cells to secrete serotonin (121). Furthermore, these microbes can synthesize neuroactive substances, including GABA and dopamine (122). As previously noted, reduced microbial diversity in depressed patients may decrease the production of essential metabolites like serotonin precursors, thereby perturbing neurotransmitter balance and energy metabolism in regions such as the PFC and limbic system, ultimately impairing emotional and cognitive processing. Conversely, stress-induced activation of the HPA axis elevates cortisol levels, which compromises intestinal permeability, immune responses, and mucosal barrier function, thus altering the microbial colonization environment (123). This process can trigger systemic low-grade inflammation, facilitating the translocation of pro-inflammatory cytokines (including IL-1β, IL-6, and TNF-α) into the central nervous system. Such infiltration disrupts glucose metabolism and mitochondrial function in emotion-related hubs like the hippocampus and ACC, exacerbating neuroinflammation and oxidative stress. Preclinical evidence further reveals that gut microbiota dysbiosis can activate the NLRP3 inflammasome pathway in the hippocampus, triggering localized neuroinflammatory responses accompanied by aberrant energy metabolism and impaired synaptic plasticity, which collectively induce depressive-like behaviors in postpartum mice (124). This suggests that the gut microbiota may directly influence the metabolic homeostasis of key brain regions by modulating neuroimmune-metabolic coupling mechanisms.

In summary, the gut microbiota not only participates in the pathophysiology of PND through the “gut-brain axis” but may also form an integrated “microbiota-metabolism-brain function” network involving metabolic disorders, neuroinflammation, and neurotransmitter imbalances in regions such as the PFC and hippocampus. Together, these factors drive the onset and progression of perinatal mood disorders. To facilitate a clearer understanding of the multifactorial etiology, the primary biological drivers—including hormonal, immunological, and microbiota-related factors—that contribute to the brain regional abnormalities are synthesized in **Figure 2**.

## 5 Advances in multi-omics research on perinatal depression

In recent years, the rapid evolution of bioinformatics has established multi-omics integration as a powerful tool for systematically unraveling the complex biological mechanisms underlying PND. These efforts involve the synthesis of multi-layered data spanning genomics, epigenetics, proteomics, and metabolomics.

### 5.1 Genomics and epigenetics

Genetic factors establish the susceptibility framework for the onset of PND. Much like MDD, PND is a psychiatric disorder governed by an intricate interplay of genetic, environmental, and psychosocial factors, characterized by its multifactorial and polygenic nature. The inaugural genome-wide association study (GWAS) focused on PPD suggested that its single nucleotide polymorphism heritability is consistent with that of MDD. Although the results did not reach traditional significance thresholds, the study demonstrated that PPD is a polygenic heritable phenotype that may harbor unique genetic components (125). Another GWAS identified a signature of DNA methylation markers capable of distinguishing AND and predicting the subsequent development of PPD (126). Furthermore, genomic evidence has highlighted CLCN7 as a potential pathogenic driver for PPD, primarily exerting its influence by altering brain connectivity patterns (127).

Beyond static sequences, individual genetic susceptibility and perinatal environmental stressors can exert long-term influence on gene expression through epigenetic modifications, such as DNA methylation, thereby modulating the function and metabolism of targeted brain regions. Severe maternal stress or trauma can have transgenerational effects, leading to premature fetal exposure to hypercortisolemic environments. Research indicates that such prenatal cortisol exposure augments HPA axis reactivity in offspring, increasing their lifetime vulnerability to psychiatric disorders (128). Specifically, infants of mothers with PPD exhibit increased methylation at the 1-F promoter of the glucocorticoid receptor gene (NR3C1), which correlates positively with elevated GR expression and heightened HPA axis reactivity (129). A study of mothers with PND in the Republic of Congo further revealed that prenatal stress broadly impacts the methylation of four pivotal HPA axis genes: CRH, CRHBP, NR3C1, and FKBP5 (130). Encouragingly, these epigenetic modifications may be reversible through therapeutic intervention. In offspring of mothers with AND who received cognitive behavioral therapy, overall DNA methylation levels decreased by 2.7%, although no significant changes were observed specifically in NR3C1 promoter methylation (131).

### 5.2 Proteomics

In the field of proteomics, investigations involving human-derived samples remain relatively scarce. However, existing studies have identified a distinct protein expression profile in the plasma of mothers with PND. These differentially expressed proteins are primarily enriched in neuro-related signaling pathways, such as those governing axon guidance, astrocyte differentiation, and the maintenance of GABAergic neurons (131). By comparing proteomic signatures in the PFC between healthy and stressed maternal rats, researchers found that the expression of αB-crystallin was most markedly elevated. As a small heat shock protein and molecular chaperone, αB-crystallin participates in neuroprotection and the repair of cellular damage; its upregulation may represent a functional compensatory mechanism to sustain neuronal hyperactivity. Another complementary study confirmed elevated levels of this protein in the rat preoptic area (POA) and found that the majority of differentially expressed proteins are involved in glucose metabolism and oxidative stress. This suggests a close metabolic association with meeting the heightened energy demands of hyperactive maternal neurons (132).

### 5.3 Metabolomics

Distinct metabolic profiles provide a critical reference for diagnosing PND and elucidating its underlying pathophysiological mechanisms. Plasma metabolomics research indicates that mothers with AND exhibit elevated glucose and lactate metabolism alongside diminished pyruvate metabolism, suggesting adaptive shifts in the tricarboxylic acid (TCA) and lactate cycles under chronic stress (133). This systemic metabolic signature likely reflects cerebral energy dysregulation; specifically, heightened glucose metabolism may represent a compensatory response to the increased energy demands of regions such as the PFC and hippocampus. Conversely, reduced pyruvate levels may impair the generation of acetyl-coenzyme A (acetyl-CoA), thereby disrupting mitochondrial function and neuronal energy homeostasis, which ultimately compromises synaptic plasticity and neural circuit integrity. Furthermore, evidence that differential metabolites in PPD patients are significantly enriched in the TCA cycle underscores the role of core energy metabolism disturbances in driving neurofunctional impairments (134).

Consistent with plasma characteristics, urinary metabolomic analyses of PPD patients have similarly revealed elevated glucose levels (135). Additionally, increased urinary concentrations of xanthine and hypoxanthine correlate positively with the severity of maternal depressive symptoms (136). As byproducts of purine catabolism, the accumulation of these metabolites may reflect imbalances in cerebral adenosine triphosphate metabolism and heightened oxidative stress, the latter of which can impair the functional capacity of the frontal lobe and limbic system. Multiple studies indicate that metabolites highly expressed in PPD—including kynurenine, kynurenic acid, and eicosapentaenoic acid—are linked to dysregulated amino acid, fatty acid, and steroid metabolic pathways (137). Notably, shifted kynurenine metabolism may deplete serotonin production. Other plasma-based investigations have identified differential metabolites such as glycerol, threonine, 2-hydroxybutyric acid, and phenylalanine, which are primarily enriched in serine/threonine and glycerophospholipid pathways (138). Dysregulation of these pathways may contribute to functional disturbances in emotion-regulating brain regions by exacerbating oxidative stress, perturbing neurotransmitter equilibrium, and compromising neuronal membrane integrity. Collectively, these multifaceted metabolic disruptions appear to play a central role in the pathogenesis of PND.

## 6 Conclusion and future perspectives

In summary, perinatal depression is not merely an “emotional issue” but may represent a psychiatric disorder characterized by structural and functional abnormalities in hormone-sensitive prefrontal-limbic brain circuitry, manifesting as diminished activity in regulatory centers and hyperactivity in emotional centers. These abnormalities result from the combined effects of genetic, neuroendocrine, immune, and environmental factors. Importantly, these neurobiological insights offer a critical bridge to clinical translation. The recognition of GABAergic dysregulation and allopregnanolone fluctuations has already paved the way for targeted fast-acting therapies, such as Brexanolone. Furthermore, the glutamatergic pathway disorders and neuroinflammatory signatures highlighted in this review suggest that future interventions targeting the glutamate-GABA balance or specific immune-metabolic hubs could provide more personalized and effective treatment options.

By providing a multi-dimensional integration of structure, function, and metabolism, this review underscores the potential of brain-based biomarkers to transition PND from subjective, symptom-based screening to objective, mechanism-driven diagnosis. This framework not only clarifies the complex “hormone-immune-gut-brain” interplay but also defines the most promising neural circuits for neuromodulation and pharmacological targeting. Future research directions should focus on antenatal depression, which remains significantly understudied compared to the postpartum period, and should be dedicated to the deep integration of multimodal technologies and the cross-disciplinary application of diverse methodologies. By leveraging spatial metabolomics and single-cell sequencing technologies, *in situ* quantification of brain region-specific metabolites and detection of subtle differences at the single-cell level can be achieved. Combined with genetic and epigenetic biomarkers, these approaches will enable more precise identification of high-risk populations, facilitate early intervention, and provide new perspectives for the comprehensive life-cycle management of perinatal mental health.

## Data Availability

The original contributions presented in the study are included in the article/supplementary material. Further inquiries can be directed to the corresponding authors.
